# Development and validation of nomogram for unplanned ICU admission in patients with dilated cardiomyopathy

**DOI:** 10.3389/fcvm.2023.1043274

**Published:** 2023-03-16

**Authors:** Xiao-Lei Li, Dilare Adi, Qian Zhao, Aibibanmu Aizezi, Munawaer Keremu, Yan-Peng Li, Fen Liu, Xiang Ma, Xiao-Mei Li, Adila Azhati, Yi-Tong Ma

**Affiliations:** ^1^ State Key Laboratory of Pathogenesis, Prevention and Treatment of High Incidence Diseases in Central Asia, Department of Cardiology, First Affiliated Hospital of Xinjiang Medical University, Urumqi, China; ^2^ Xinjiang Key Laboratory of Cardiovascular Disease, Clinical Medical Research Institute, First Affiliated Hospital of Xinjiang Medical University, Urumqi, China; ^3^ The Emergency Center, First Affiliated Hospital of Xinjiang Medical University, Urumqi, China

**Keywords:** dilated cardiomyopathy, unplanned ICU admission, prognosis, nomogram, prediction model

## Abstract

**Objective:**

Unplanned admission to the intensive care unit (ICU) is the major in-hospital adverse event for patients with dilated cardiomyopathy (DCM). We aimed to establish a nomogram of individualized risk prediction for unplanned ICU admission in DCM patients.

**Methods:**

A total of 2,214 patients diagnosed with DCM from the First Affiliated Hospital of Xinjiang Medical University from January 01, 2010, to December 31, 2020, were retrospectively analyzed. Patients were randomly divided into training and validation groups at a 7:3 ratio. The least absolute shrinkage and selection operator and multivariable logistic regression analysis were used for nomogram model development. The area under the receiver operating characteristic curve, calibration curves, and decision curve analysis (DCA) were used to evaluate the model. The primary outcome was defined as unplanned ICU admission.

**Results:**

A total of 209 (9.44%) patients experienced unplanned ICU admission. The variables in our final nomogram included emergency admission, previous stroke, New York Heart Association Class, heart rate, neutrophil count, and levels of N-terminal pro b-type natriuretic peptide. In the training group, the nomogram showed good calibration (Hosmer–Lemeshow *χ*^2 ^= 14.40, *P *= 0.07) and good discrimination, with an optimal-corrected C-index of 0.76 (95% confidence interval: 0.72–0.80). DCA confirmed the clinical net benefit of the nomogram model, and the nomogram maintained excellent performances in the validation group.

**Conclusion:**

This is the first risk prediction model for predicting unplanned ICU admission in patients with DCM by simply collecting clinical information. This model may assist physicians in identifying individuals at a high risk of unplanned ICU admission for DCM inpatients.

## Introduction

Dilated cardiomyopathy (DCM) is characterized by dilatation and impaired function of ventricles (1). Studies have shown that 5–8.34 cases of DCM occur per 100,000 people per year, with the 5-year survival rate of only 50% (2, 3). The incidence of adverse events is an important factor that affects the prognosis of patients with DCM (4, 5). Unplanned intensive care unit (ICU) admission is the major in-hospital adverse event for DCM inpatients. Compared to direct ICU admission, unplanned ICU admission is associated with poorer in-hospital prognosis and substantially mortality rates (6, 7). Additionally, unplanned ICU admission can significantly magnify the psychological stress of patients and their families (8). Therefore, assessing risks from unplanned ICU admission is not only just for managing individualization prognosis but also for improving healthcare quality.

According to the reports, approximately 36% of unplanned ICU admission is preventable; therefore, early identification can effectively improve patient survival and rationalize the use of healthcare resources (9–11). Several scoring systems, such as Early Warning Scores (EWS) and National Early Warning Score (NEWS), have been widely developed and used for identifying patients at risk of early disease progression (12, 13); however, most risk prediction models are established based on general emergency patients, and the generic prediction model fails to fit the featured population (14). In DCM inpatients, to the best of our knowledge, no prediction models have been developed for assessing unplanned ICU admission.

In this study, we retrospectively analyzed 2,214 inpatients with DCM and without planned ICU admission at baseline and aimed to develop a nomogram for individualized prediction of unplanned ICU admission incidents in DCM patients by simply collecting clinical information.

## Materials and methods

### Study design and population

This study included 2,735 DCM patients from the retrospective cohort study, which was designed to evaluate the clinical outcomes and risk factors of cardiomyopathy, and the detailed protocol has been registered on www.chictr.org.cn (registration number: ChiCTR2200058051). This registration trial included 5,937 patients with primary cardiomyopathy, namely, DCM, hypertrophic cardiomyopathy (HCM), restrictive cardiomyopathy (RCM), arrhythmogenic right ventricular cardiomyopathy (ARVC), and unclassified cardiomyopathy, and the diagnostic criteria refer to the JCS/JHFS 2018 Guideline on the Diagnosis and Treatment of Cardiomyopathies (3). In addition, this registration trial excluded patients who had malignant tumors, hematological malignancy, autoimmune diseases, serious dysfunction of the kidney or liver, pregnant or lactating women, and patients younger than 18 years old. All patients were admitted to the First Affiliated Hospital of Xinjiang Medical University from January 01, 2010, to December 31, 2020, and the data were obtained from electronic medical records and follow-up.

**Figure 1:**
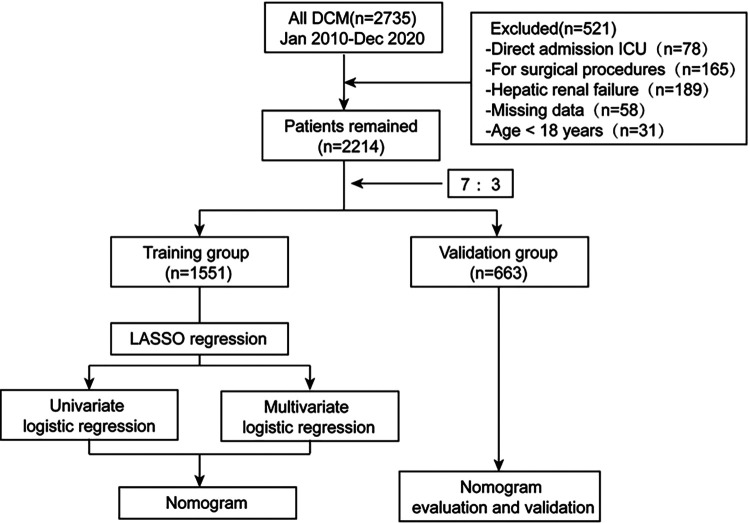
Flow diagram of the study.

To investigate the individualized risk of unplanned ICU admission in DCM inpatients, a total of 2,735 patients were initially evaluated and 521 were excluded, leading to ultimately 2,214 patients in this study, of which 209 experienced unplanned ICU admission (ICU+) and 2005 were not admitted to the ICU (ICU−). Patient selection and study flow are shown in Figure 1. The inclusion criteria for the present study were as follows (15): (1) left ventricular end-diastolic dimension (LVEDD) >5.0 cm in females and >5.5 cm in males; and (2) left ventricular ejection fraction (LVEF) <45%. We excluded patients who (1) had ischemic heart disease, hypertensive heart disease, valvular heart disease, or congenital heart disease; (2) had direct admission to ICU; (3) had severe hepatic and renal failure; (4) were admitted for surgical procedures; (5) were younger than 18 years; and (6) had incomplete clinical information. Finally, 2,214 eligible patients were included in this study. Electronic medical records were fully reviewed by two independent reviewers according to the inclusion and exclusion criteria. All study personnel had formal training prior to participation in the study.

### Data collection

All data were obtained from the first measurement at admission. Demographic data, comorbidities, blood tests, and echocardiographic results were included for all patients. Comorbidities included hypertension, diabetes mellitus, stroke, atrial fibrillation (AF), and chronic obstructive pulmonary disease (COPD).

Patients who reported smoking in the previous 6 months were considered current smokers. Similarly, patients who consumed alcohol in the last half a year were considered current drinkers. Hypertension was defined as patients with at least three resting measurements above 140/90 mmHg taken from at least two separate healthcare visits or history of hypertension with active treatment, as suggested by the American Heart Association (16). Diabetes was defined as having a history of diabetes with using hypoglycemic drugs or random intravenous plasma glucose of 200 mg/dL (11.1 mmol/L), or 2-h plasma glucose of 200 mg/dL (11.1 mmol/L) after an oral glucose tolerance test (OGTT), fasting blood glucose (FPG) of 126 mg/dL (7.0 mmol/L), or hemoglobin A1c (HbA1c) of 6.5% (17). Stoke was diagnosed by magnetic resonance imaging (MRI) combined with clinical neurological dysfunction (18) and a patient known to had stroke prior to this visit was categorized as prior stroke. AF included all types of previously diagnosed AF, including paroxysmal AF, persistent AF, and permanent AF (19). COPD was defined as a disease characterized by persistent respiratory symptoms and airflow limitation and diagnosed by the Global initiative for chronic Obstructive Lung Disease (GOLD) (20). Severe renal insufficiency was defined as estimated glomerular filtration rate (eGFR) <30 mL/min (21). Severe hepatic insufficiency is defined as alanine transaminase (ALT) or aspartate aminotransferase (AST) exceeding the upper limit of normal by a factor of 5, specifically AST >180 U/L and ALT >260 U/L (22).

### Outcome assessment

The primary outcome was unplanned ICU admission, which was defined as transfer to the ICU due to deterioration or developed complications (23).

### Statistical analysis

All statistical analysis were performed using Social Package for the Social Sciences (SPSS) version 26.0 (SPSS Inc., Chicago, IL, United States), and R software version 4.0.3 (https://cran.r-project.org). The R software mainly include package of “glmnet,” “caret,”“rms,” “pROC,” “rmda,” and so on.

### Division of datasets

A total of 2,214 DCM patients were randomly divided into two groups, the training group (*n* = 1,551) and the validation group (*n* = 663), at a theoretical ratio of 7 : 3.

### Variable selection

The least absolute shrinkage and selection operator (LASSO) regression is an efficient statistical method to filter out the most important features from high-dimensional data. We performed LASSO regression in the training group to screen out the most useful predictor variables for unplanned ICU admissions. The nonzero coefficient characteristic variables corresponding to the maximum *λ* within 1 standard deviation (SD) of the mean error was the final model predictor variables.

### Model development and validation

The risk prediction model was developed by multivariate logistic regression, where the dependent variable in the model was unplanned ICU admission, while the independent variables included predictors selected from the LASSO regression. To provide clinicians with a quantitative tool to predict the risk of unplanned ICU admissions, we constructed a nomogram based on multivariate logistic regression analysis. Nomogram performance was evaluated by both discriminations, presented as C-index and the area under the receiver operating characteristic curve (AUC), and calibration, expressed as the Hosmer–Lemeshow test and calibration plot. Discrimination and calibration were also accounted for in estimating the validity of the model in the validation group. To assess the clinical validity of the model, the decision curve analysis (DCA) and clinical impact curve (CIC) were constructed, which were mainly quantitative analyses of the net returns under different threshold probabilities.

Categorical variables were expressed as frequencies (%) and continuous variables were expressed as mean ± SD or median (interquartile range). The differences in baseline characteristics between the two groups were examined by independent-samples *t*-test or Mann–Whiney *U*-test for continuous variables and the Pearson chi-square test (Pearson *χ*^2^ test) or Fisher exact test for categorical variables, as appropriate. All tests were two-sided, and a *P* value <0.05 was considered statistically significant.

## Result

### Patient characteristics in the training group

A total of 1,551 (male, 73.1%) and 663 patients (male, 72.39%) with DCM comprised the training and validation groups, respectively. There were 147 (9.47%) and 62 (9.35%) patients who had unplanned ICU admission in the training and validation groups, respectively. As shown in Table 1, compared with the ICU− patients, ICU+ patients, have a higher ratio of emergency admission (EA), *β* blocker usage, New York Heart Association (NYHA) class, heart rate, white blood cell count, neutrophil count, aspartate aminotransferase, higher N-terminal pro b-type natriuretic peptide, larger right ventricular diameter (RV), and larger right atrial diameter (RA) (all *P *< 0.05, respectively); the lymphocyte count, monocyte count, fasting blood glucose, serum albumin, serum sodium, and serum potassium were significantly lower than those in the ICU− group (all *P *< 0.05, respectively).

**Table 1:** Baseline characteristics of patients in the training and validation group.

Variable	Total	Training group		*P* value	Validation group		*P* value
		ICU−	ICU+		ICU−	ICU+	
		(*n* = 1,404)	(*n* = 147)		(*n* = 601)	(*n* = 62)	
Age (years)	54 (45–63)	54 (45–63)	53 (43–61.5)	**0**.**122**	55 (44–62)	54 (44–62)	**0**.**787**
Male, *n* (%)	1,609 (72.7)	1,026 (73.1)	103 (70.1)	**0**.**435**	433 (72.0)	47 (75.8)	**0**.**528**
**Ethnicity, *n* (%)**
Han	1,133 (51.2)	720 (51.3)	78 (53.2)	**0**.**902**	305 (50.7)	30 (48.4)	**0**.**913**
Uygur	706 (31.9)	458 (32.6)	47 (31.9)	** **	182 (30.3)	19 (30.6)	** **
Other races	375 (16.9)	226 (16.1)	22 (14.9)	** **	114 (19.0)	13 (21.0)	** **
**Admission form, *n* (%)**
Emergency	620 (28.0)	379 (27.0)	70 (47.6)	**<0**.**001**	141 (23.5)	30 (48.4)	**<0**.**001**
Referral	454 (20.5)	285 (20.3)	25 (17.0)	** **	132 (22)	12 (19.4)	** **
Clinic	1,140 (51.5)	740 (52.7)	52 (35.4)	** **	328 (54.6)	20 (32.2)	** **
Smoking, *n* (%)	841 (37.9)	547 (39.0)	55 (37.4)	**0**.**715**	217 (36.1)	22 (35.5)	**0**.**923**
Drinking, *n* (%)	495 (22.4)	325 (23.1)	33 (22.4)	**0**.**848**	125 (20.8)	12 (19.4)	**0**.**789**
**NYHA, *n* (%)**
Grade II–III	1,729 (78.1)	1,120 (79.8)	84 (57.1)	**<0**.**001**	493 (82.0)	32 (51.6)	**<0**.**001**
Grade IV	485 (21.9)	284 (20.2)	63 (42.9)	** **	108 (18.0)	30 (48.4)	** **
Weight (kg)	75 (63,85)	74.5 (63,84)	75 (63,83)	**0**.**906**	75 (63,86)	74.5 (64.2,85)	**0**.**619**
SBP (mmHg)	120 (105–130)	120 (105–130)	118 (104.5–130)	**0**.**531**	120 (105–130)	111 (102–123.8)	**0**.**043**
DBP (mmHg)	76 (67–84)	75 (68–83)	75 (68.5–85.5)	**0**.**369**	76 (67–85)	75 (65.2–80)	**0**.**318**
HR (beats/min)	84 (76–99)	84 (76–98)	96 (78–110)	**<0**.**001**	84 (76–98)	90 (78.5–99.8)	**0**.**104**
**Medical history, *n* (%)**
Hypertension	666 (30.1)	428 (30.5)	42 (28.6)	**0**.**631**	183 (30.4)	13 (20.9)	**0**.**119**
Diabetes mellitus	311 (14.1)	196 (14.0)	24 (16.3)	**0**.**434**	79 (13.1)	12 (19.4)	**0**.**176**
Previous stroke	107 (4.8)	64 (4.6)	20 (13.6)	**<0**.**001**	19 (3.2)	4 (6.5)	**0**.**26**
AF	331 (15.1)	213 (15.2)	27 (18.4)	**0**.**308**	80 (13.3)	11 (17.7)	**0**.**334**
COPD	158 (7.1)	110 (7.8)	9 (6.1)	**0**.**458**	34 (5.7)	5 (8.1)	**0**.**398**
**Laboratory characteristics**
WBC (10^9^/L)	6.95 (5.68–8.44)	6.80 (5.60–8.30)	7.60 (6.30–9.40)	**<0**.**001**	7.00 (5.70–8.40)	7.70 (6.40–9.90)	**0**.**003**
Neut (10^9^/L)	4.28 (3.32–5.59)	4.20 (3.30–5.50)	4.90 (3.90–6.80)	**<0**.**001**	4.30 (3.30–5.50)	5.20 (4.10–7.40)	**<0**.**001**
Lymph (10^9^/L)	1.74 (1.34–2.25)	1.80 (1.40–2.30)	1.60 (1.30–2.20)	**0**.**032**	1.80 (1.40–2.30)	1.40 (1.00–1.90)	**<0**.**001**
Mono (10^9^/L)	0.54 (0.42–0.70)	0.50 (0.40–0.70)	0.60 (0.50–0.80)	**<0**.**001**	0.50 (0.40–0.70)	0.70 (0.50–0.80)	**<0**.**001**
Hb (g/L)	140 (127–152)	140 (127–152)	140 (126.5–154)	**0**.**847**	139 (127–152)	135 (116.8–147.8)	**0**.**082**
Serum creatinine (umol/L)	80.00 (66.84–95.83)	80.00 (67.00–95.80)	83.00 (66.80–101.60)	**0**.**238**	79.00 (65.10–94.00)	90.90 (72.20–105.90)	**0**.**006**
Serum urea (mmol/L)	6.40 (5.10–8.00)	6.40 (5.10–8.00)	6.50 (5.20–8.40)	**0**.**432**	6.20 (5.10–7.90)	6.80 (5.50–8.00)	**0**.**042**
ALT (U/L)	26.50 (17.80–44.32)	26.50 (17.40–43.80)	26.00 (18.20–49.70)	**0**.**428**	26.50 (17.90–43.20)	28.50 (19.50–53.50)	**0**.**118**
AST (U/L)	25.41 (18.90–35.60)	25.00 (18.70–34.30)	29.00 (21.50–40.40)	**<0**.**001**	24.70 (18.60–36.00)	28.70 (21.00–48.10)	**0**.**024**
Serum albumin (g/L)	37.10 (33.40–40.59)	37.20 (33.60–40.50)	35.3 (31.10–39.70)	**<0**.**001**	37.30 (33.70–40.70)	35.10 (31.70–39.20)	**0**.**02**
Serum sodium (mmol/L)	3.83 (3.54–4.16)	3.80 (3.50–4.20)	3.80 (3.50–4.20)	**0**.**819**	3.84 (3.54–4.15)	3.78 (3.45–4.28)	**0**.**905**
Serum potassium (mmol/L)	140.00 (137.08–142.50)	140.20 (137.50–142.7)	138.40 (135.60–141.00)	**<0**.**001**	140.00 (137.20–142.60)	137.70 (134.10–139.80)	**<0**.**001**
Serum chloride (mmol/L)	104.00 (101.00–106.60)	104.00 (101.10–106.60)	103.90 (100.30–106.60)	**0**.**485**	104.00 (101.20–106.50)	102.80 (98.60–106.10)	**0**.**017**
Serum calcium (mmol/L)	2.21 (2.13–2.30)	2.20 (2.10–2.30)	2.20 (2.10–2.30)	**0**.**484**	2.20 (2.10–2.30)	2.20 (2.10–2.30)	**0**.**047**
FBG (mmol/L)	5.54 (4.74–7.04)	5.50 (4.70–6.90)	5.90 (5.00–7.30)	**0**.**004**	5.50 (4.80–7.00)	6.20 (5.10–7.90)	**0**.**013**
NT-proBNP/100 (ng/mL)	23.52 (9.76–50.27)	21.60 (8.90–47.60)	42.90 (18.30–88.90)	**<0**.**001**	20.70 (9.60–42.40)	52.70 (27.40–87.40)	**<0**.**001**
**Echocardiography characteristics**
LA (mm)	44 (40–49)	44 (40–49)	45 (40–49)	**0**.**451**	44 (41–49)	45.5 (41.2–50.5)	**0**.**331**
LVEDD (mm)	67 (62–73)	67 (62–73)	68 (63–74)	**0**.**204**	67 (62–74)	69 (64–75)	**0**.**078**
LVESD (mm)	55 (50–61.25)	55 (50–61)	57 (51–64)	**0**.**152**	55 (50–62)	57 (53–64)	**0**.**029**
RA (mm)	40 (35–46)	40 (35–46)	42 (37–48)	**0**.**003**	39 (35–46)	40 (35–49)	**0**.**306**
RV (mm)	21 (19–24.25)	21 (19–24)	22 (20–25)	**0**.**008**	21 (19–25)	22 (19–25)	**0**.**279**
LVEF (%)	35.00 (30.36–40.00)	35.50 (31.00–40.00)	35.00 (29.50–39.00)	**0**.**128**	35.00 (30.00–40.00)	33.00 (30.00–37.80)	**0**.**087**
**Medication, *n* (%)**
ACEI/ARB	1,462 (66.0)	930 (66.2)	93 (63.3)	**0**.**469**	406 (67.6)	33 (53.2)	**0**.**023**
*β* blocks	1,614 (72.9)	1,043 (74.3)	97 (66.0)	**0**.**03**	433 (72.0)	41 (66.1)	**0**.**326**
MRA	1,729 (78.1)	1,110 (79.1)	106 (72.1)	**0**.**051**	468 (77.9)	45 (72.6)	**0**.**343**
Diuretic	1,172 (52.9)	745 (53.1)	74 (50.3)	**0**.**529**	320 (53.2)	33 (53.2)	**0**.**998**
Digoxin	849 (38.3)	543 (38.7)	46 (31.3)	**0**.**079**	367 (61.1)	36 (58.1)	**0**.**645**
**Instrumentation, *n* (%)**
CRT/CRTD	90 (4.1)	1,349 (96.1)	138 (93.9)	**0**.**201**	23 (3.8)	3 (4.8)	**0**.**727**
ICD	55 (2.5)	35 (2.5)	6 (4.1)	**0**.**253**	12 (2.0)	2 (3.2)	**0**.**383**

Bold represents the categories of the Variable.

ICU, intensive care unit; ICU+, unplanned ICU admission (ICU+); ICU−, not admitted to the ICU; NYHA, New York Heart Association; SBP, systolic blood pressure; DBP, diastolic blood pressure; HR, heart rate; AF, atrial fibrillation; COPD, chronic obstructive pulmonary disease; WBC, white blood cell count; Lymph, lymphocyte count; Neut, neutrophil count; Mono, monocyte count; Hb, hemoglobin; AST, aspartate aminotransferase; ALT, alanine transaminase; FBG, fasting blood glucose; NT-proBNP, N-terminal pro b-type natriuretic peptide; LA, left atrial diameter; LVEDD, left ventricular end-diastolic diameter; LVESD, left ventricular end-systolic diameter; RV, right ventricular diameter; RA, right atrial diameter; LVEF, left ventricular ejection fraction; ACEI, angiotensin converting enzyme inhibitor; ARB, angiotensin receptor blockers; MRA, mineralocorticoid receptor antagonist; CRT, cardiac resynchronization therapy; CRTD, cardiac resynchronization therapy defibrillator; ICD, implantable cardioverter-defibrillator.

Patients in the ICU+ group were more likely to have larger RA and RV (all *P* < 0.05), and the left atrial diameter (LA), LVEDD, left ventricular end-systolic diameter (LVESD), and LVEF had no significant difference between the two groups.

### Variable selection

**Figure 2:**
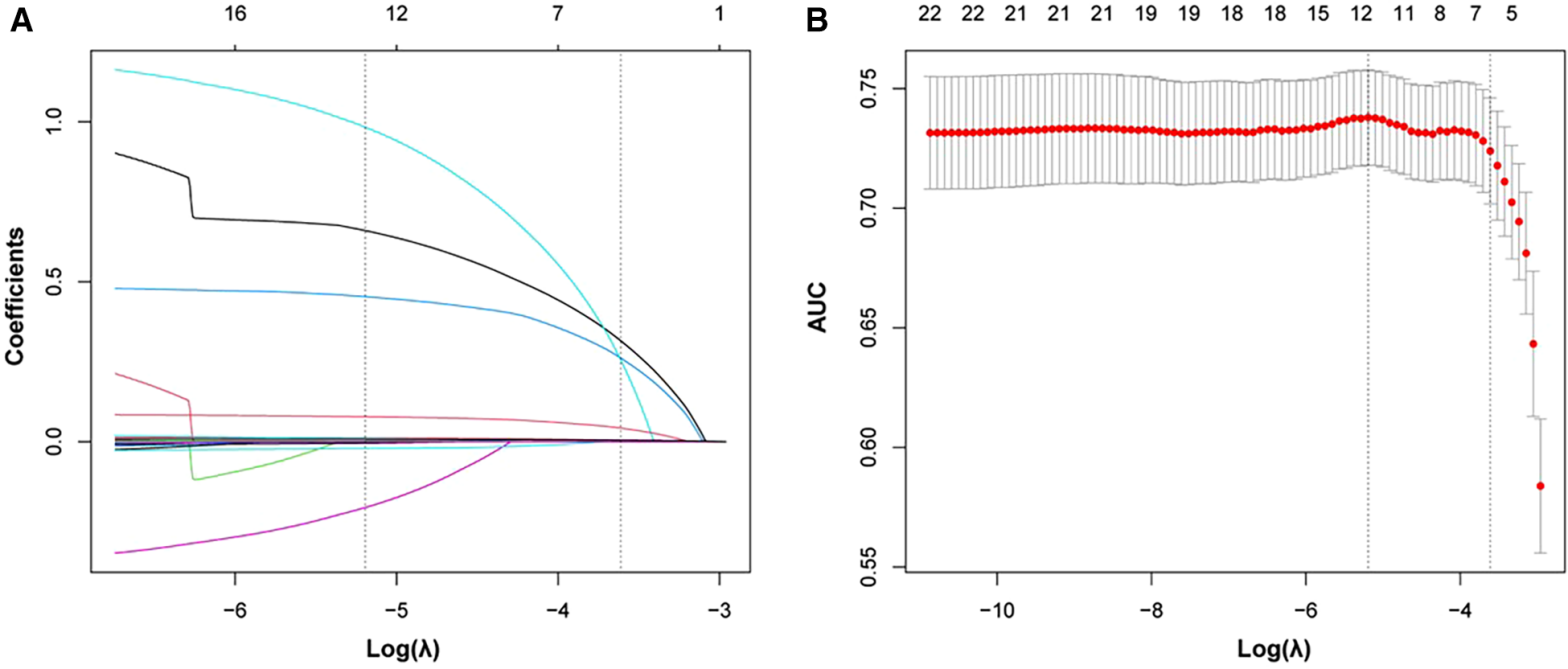
Significant variables selection using the LASSO. **(A)** Plot of each variable’s coefficient profile against log(lambda). **(B)** Ten-fold cross-validation used to validate the optimal lambda in the LASSO model. LASSO, least absolute shrinkage and selection operator.

In order to select variables that could predict the primary outcome, we included prespecified variables and variables that were statistically different between two groups into the LASSO regression analysis. The prespecified variables were selected based on clinical experience and current literature reports, as well as consensus on DCM prognostic stratification (24, 25). They were age, LVEDD, LVESD, and LVEF. Finally, we selected six statistically significant variables including emergency admission, previous stroke, NYHA class, heart rate, neutrophil count, and N-terminal pro b-type natriuretic peptide (NT-proBNP)/100 (Figure 2).

### Model development

In order to simplify the model and make it easier to use, based on optimal cutoff values, we converted heart rate (100 beats/min) and neutrophil count (4.385 × 10^9^/L) into classified variables. Then, we used logistic regression analysis to analyze the incidence of unplanned ICU admission of DCM patients in the training group, finding that ED [odds ratio (OR): 2.13; 95% confidence interval (CI): 1.48–3.06, *P *< 0.001], previous stroke (OR: 3.12, 95% CI: 1.76–5.55, *P < *0.001), NYHA class IV (OR: 1.81, 95% CI: 1.23–2.65, *P *= 0.002), heart rate (OR: 2.63, 95% CI: 1.54–3.33, *P *< 0.001), neutrophil count (OR: 1.90, 95% CI: 1.31–2.76, *P *= 0.001), and NT-proBNP/100 (OR: 1.01, 95% CI: 1.01–1.01, *P *< 0.001) were independent risk factors for unplanned ICU admission in DCM patients (*P *< 0.001) (Table 2).

**Table 2:** Logistic regression analysis of predictors of unplanned ICU admission.

Variables	*β*	Univariate analysis			*β*	Multivariable analysis		
		OR	95% CI	*p* value		OR	95% CI	*p* value
Emergency admission	1.06	2.90	2.05–4.09	<0.001	0.76	2.13	1.48–3.06	<0.001
Previous stroke	1.19	3.30	1.93–5.63	<0.001	1.14	3.12	1.76–5.55	<0.001
NYHA class	1.08	2.96	2.08–4.20	<0.001	0.59	1.81	1.23–2.65	0.002
HR	1.15	3.16	2.20–4.53	<0.001	0.82	2.63	1.54–3.33	<0.001
Neut	0.87	2.39	1.68–3.41	<0.001	0.64	1.90	1.31–2.76	0.001
NT-proBNP/100	0.01	1.01	1.01–1.02	<0.001	0.01	1.01	1.01–1.01	<0.001

ICU, intensive care unit; OR, odds ratio; 95% CI, 95% confidence interval; NYHA, New York Heart Association; HR, heart rate; Neut, neutrophil count; NT-proBNP, N-terminal pro b-type natriuretic peptide.

### Nomogram model display

**Figure 3:**
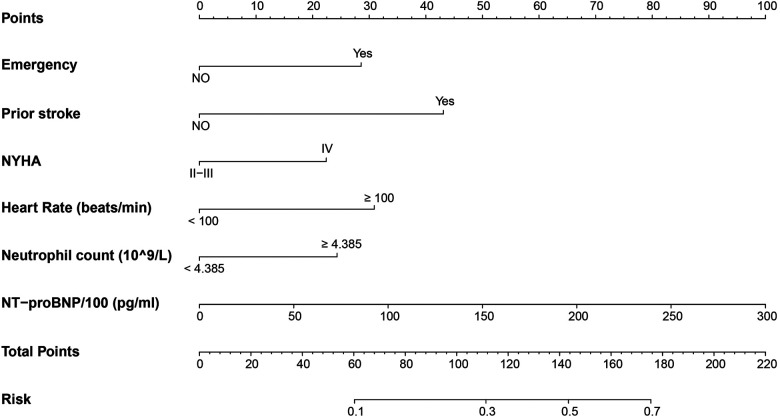
Nomogram to predict the risk of unplanned ICU admission in DCM inpatients. Points were assigned for each variable by drawing a line upward from the corresponding values to the “points line.” The “total points” was calculated as the sum of the individual score of each of the six variables included in the nomogram. We can estimate the risk of unplanned ICU admission for this patient by the probability corresponding to the “total points.” ICU, intensive care unit; DCM, dilated cardiomyopathy; NYHA, New York Heart Association; NT-proBNP, N-terminal pro b-type natriuretic peptide.

Six independent risk variables were used to build a nomogram for predicting the risk of unplanned ICU admission in patients with DCM. The scores corresponding to each predictor variable in the nomogram were summed, and the resulting probability value corresponding to the total score is the probability of risk of unplanned ICU admission (Figure 3).

### Nomogram evaluation and validation

**Figure 4:**
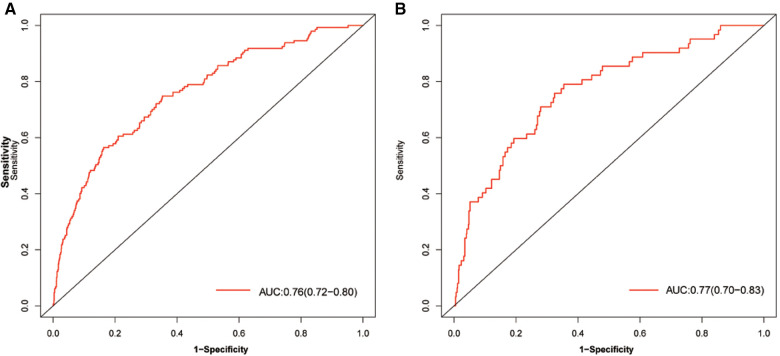
AUC of the model for predicting unplanned ICU admission of DCM patients. A, Development group. B, Validation group. Red curve shown the receiver operating characteristic curve (ROC) for nomogram. AUC, area under curve.

**Figure 5:**
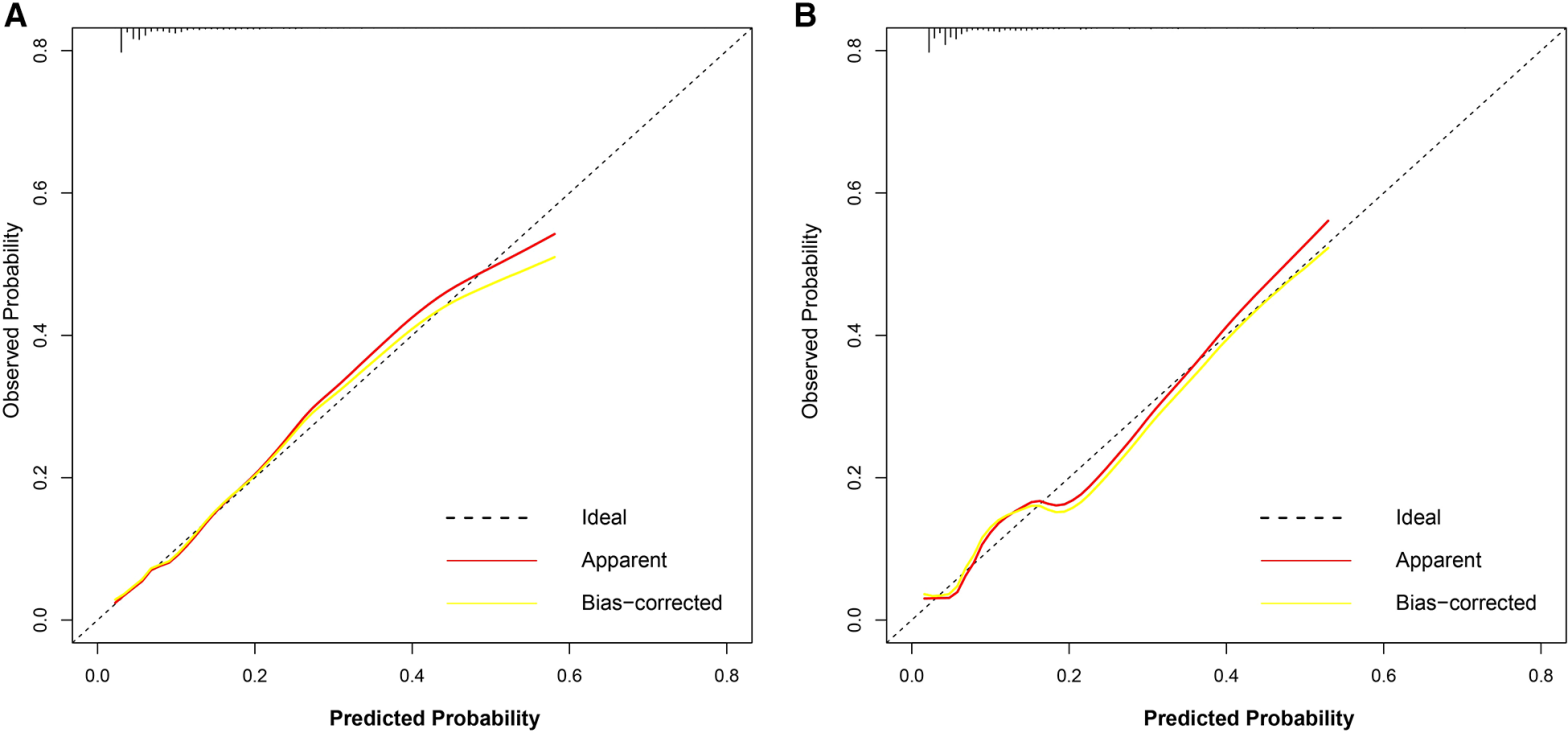
Calibration curve of the nomogram for the development group (**A**) and the validation group (**B**). The dotted line represents the ideal prediction, while the red line represents the actual calibration curve of the nomogram. The yellow line meanwhile represents the internal corrected calibration curve of the nomogram.

The discriminatory ability of the nomogram was evaluated by calculating the C-statistic as 0.76 (95% CI: 0.72–0.80) in the training group. The corrected C-statistic from bootstrap resampling showed good internal validation with a value of 0.75. The model proved to be accurate in predicting unplanned ICU admissions of DCM patients with an AUC of 0.76 (95% CI: 0.72–0.80, Figure 4A). The Hosmer–Lemeshow test showed that the model has good calibration (*χ*^2 ^= 14.40, *P *= 0.07), and the calibration curves similarly showed good calibration between the predicted and actual risk of unplanned ICU admissions for DCM patients (Figure 5A).

The C-index also reached 0.78 in the validation group and the AUC was 0.77 (95% CI: 0.70–0.83), as shown in Figure 4B. The calibration curves of the nomogram also suggested a good agreement between the actual and the predicted outcomes (Figure 5B).

**Figure 6:**
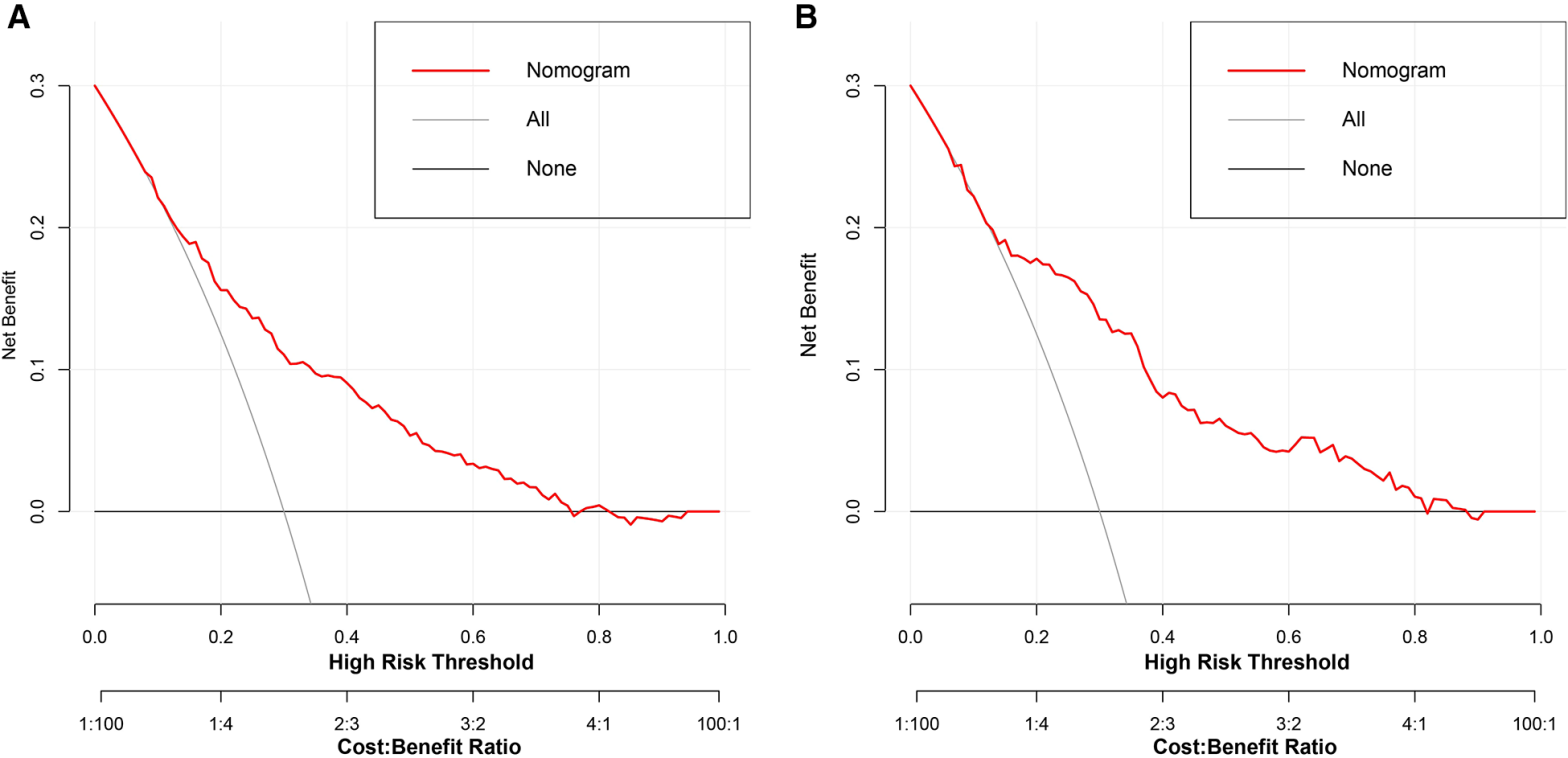
Decision curve analysis of the nomogram for the development group (**A**) and the validation group (**B**). The black line indicates that for extreme cases, the model predicts that all DCM patients have low-ICU+ probability and the clinical net benefit is 0. The gray curve indicates that for extreme cases, the model predicts that all DCM patients have moderate or high ICU+ probability and the clinical net benefit is the negative slope. The red line indicates that the model has clinical net benefit. The red line is higher than the gray and black lines, indicating that patients can benefit from the model. ICU, intensive care unit; DCM, dilated cardiomyopathy.

**Figure 7:**
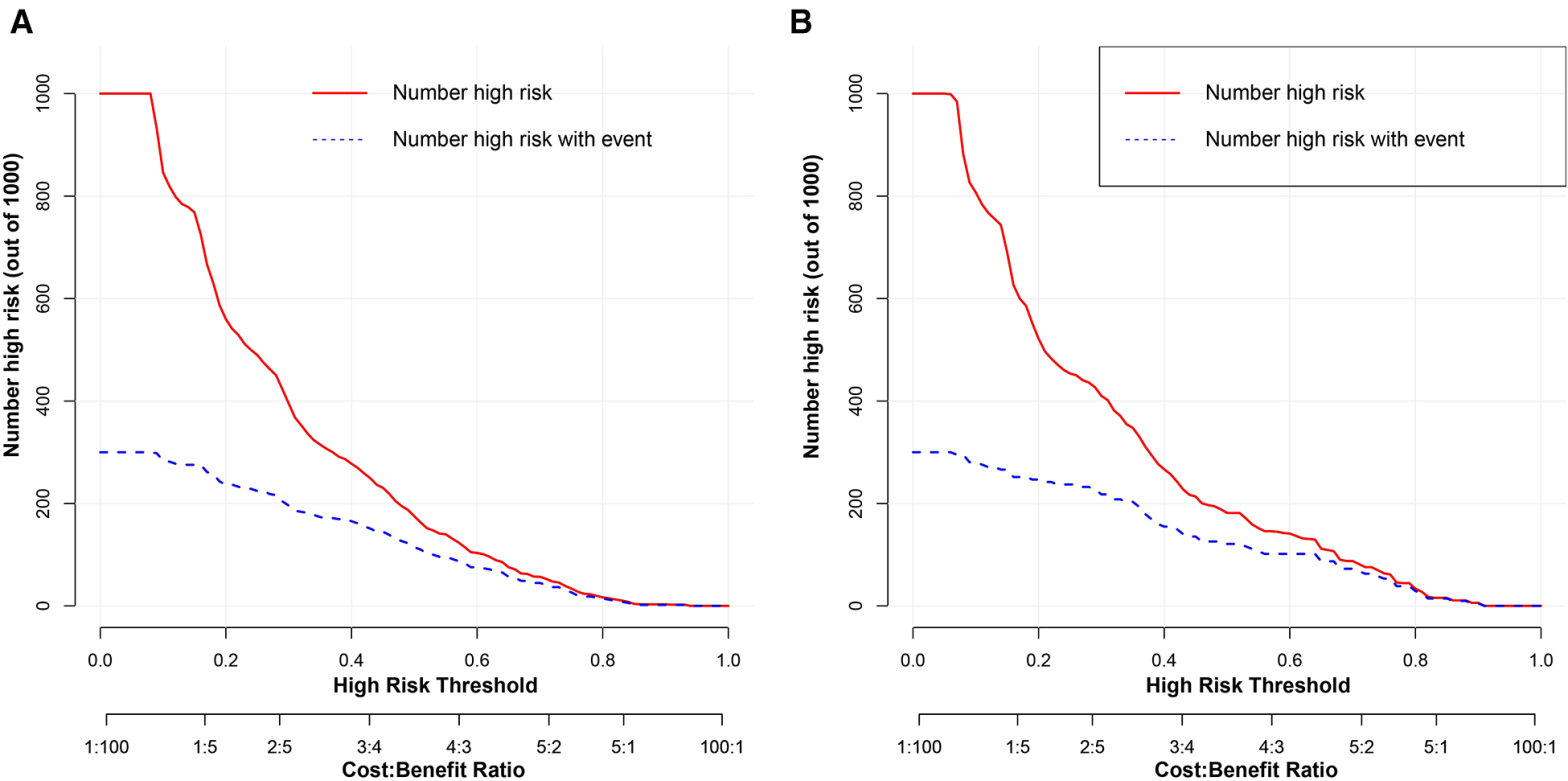
Clinical impact curve analysis of the nomogram for development group (**A**) and the validation group (**B**). The red curve indicates the number of people classified as positive (high risk) by the model at each threshold probability, and the blue curve is the number of true positives.

To estimate the clinical utility of the nomogram, DCA and CIC were used. The results of DCA are presented in Figure 6, showing that the use of this model for making clinical decisions has more benefit than the “no intervention” or “all intervention” scenarios when the unplanned ICU admission threshold probability was between 0 and 0.75 in the training group and was generally between 0.0 and 1 in the validation group. Also, CIC analysis showed the clinical efficiency of the nomogram, when the threshold probability was greater than 65%; the prediction model determined that the population at high risk for unplanned ICU admission highly matched the population experiencing unplanned ICU admissions (Figure 7).

### Performance of the GWTG-HF score of DCM inpatients

**Figure 8:**
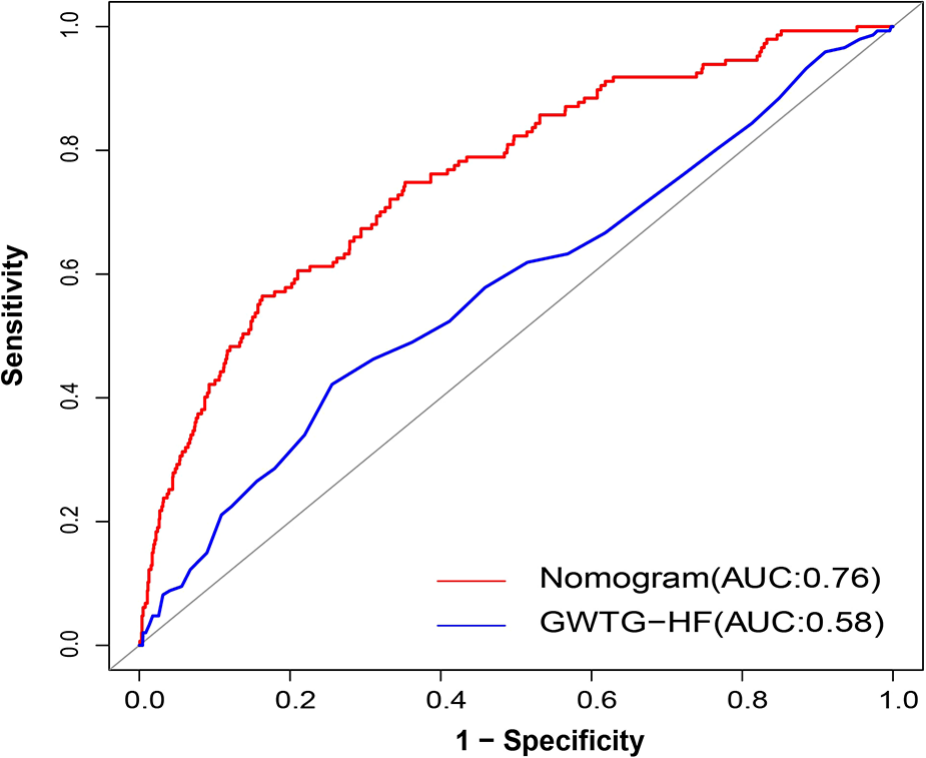
AUC of nomogram and GWTG-HF. Red and blue curves shown the ROC for nomogram and GWTG-HF. AUC, area under curve; GWTG-HF, Get With the Guidelines-Heart Failure; ROC, receiver operating characteristic curve.

The data showed that the Get With the Guidelines-Heart Failure (GWTG-HF) score was an independent risk factor for unplanned ICU admission in DCM patients (OR: 1.04; 95% CI: 1.02–1.06; *P *< 0.001). In parallel, we evaluated the ability of the GWTG-HF score to assess the risk of unplanned transfer to the ICU for DCM inpatients. However, the diagnostic power of the GWTG-HF score was general, and the AUC of unplanned ICU admission was only 0.58 (95% CI: 0.53–0.63). The DeLong test suggested a statistically significant difference between the nomogram model and the GWTG-HF score in the ability to differentiate patients with high risk of unplanned ICU admission (*P *< 0.001, Figure 8).

## Discussion

Early identification of DCM inpatients at high risk of unplanned ICU admission provides an important opportunity to assess deterioration and make timely changes in the treatment strategy. To overcome this practical need, for the first time we developed and validated a nomogram for DCM inpatients to predict the risk of unplanned ICU admissions. Emergency admission, previous stroke, NYHA class, heart rate, neutrophil count, and NT-proBNP were demonstrated to be predictors of elevated risk for unplanned ICU admissions.

Previous studies have reported that, in general internal inpatients, unplanned ICU admissions contribute to 14%–28% of ICU admissions (12, 26). From our data, the percentage of unplanned ICU admissions occurring is roughly 9.44% (209/2214) among DCM inpatients, and most of these inpatients were admitted through ED (47.85%, 100/209). In our study, the admission pathway was a valuable predictor of deterioration (OR: 2.13, 95% CI: 1.48–3.06, *P *< 0.001). Numerous studies have also confirmed that patients transferred from the general unit to ICU for intensive care management have higher in-hospital mortality than those admitted directly from ED (7, 9). Therefore, we considered that advance risk assessment and management of DCM patients with emergency admissions might be an effectively managed approach. Currently, the Medical Emergency Team (MET) system could be widely applied and extended to assess the risk of an emergency patient. Efficient use of the MET system can reduce the incidence of unplanned ICU admissions and is independently associated with reduced hospital mortality (27, 28).

Of note, some studies have been conducted on the risk assessment of unplanned ICU admissions. At present, the EWS has been used internationally and is widely promoted (29, 30). The NEWS, established by the Royal College of Physicians of London in 2012, is one of the scoring systems used to assess the severity of acute illnesses (13). The NEWS has proven to be a very effective tool for assessing the risk of in-hospital adverse events such as unplanned ICU admissions and in-hospital deaths. The predictors included in the NEWS were respiration rate, SpO_2_, any supplemental oxygen, temperature, SBP, heart rate, and level of consciousness, among which respiration rate, SpO_2_, and heart rate affect unplanned ICU admissions. Also, heart rate was the most important predictor in our nomogram model, emphasizing the importance of vital signs in assessing the risk of unplanned ICU admissions. Lindgren et al. showed the causal relationship between increased heart rate and myocardial systolic dysfunction, and fast heart rate in adolescents was strongly associated with the development of DCM-related heart failure (31). The TRED-HF study also found that increased heart rate might be a valid indicator of worsening cardiac function and relapse in patients recovering from DCM (32). Unfortunately, due to the missing arterial blood gas and temperature data, we were unable to assess the performance of the NEWS in DCM patients.

As we know, composite clinical endpoints, including unplanned ICU admission, cardiac arrest, and in-hospital mortality, were the observed outcomes for prior scores (32–35), and most of the study information was based on health records of general inpatients (33–35). Generalizing these scores to risk stratification of cardiovascular disease, therefore, may be somewhat limited. In view of the following, we formulated a new nomogram model to assess the potential risk of unplanned ICU admissions for DCM inpatients based on clinical information and laboratory characteristics. The C-index of our nomogram in the training group and the validation group were 0.76 and 0.78, respectively, indicating that the model had high discriminative power. Moreover, the calibration curve also suggested good agreement between the actual probabilities and the predicted probabilities in the training and validation groups. Our nomogram model still has a number of unique advantages. First, the population enrolled in this study differs from previous studies, which have mostly studied patients in the emergency department. In contrast, our study focused on inpatients with DCM. Second, we used LASSO regression to effectively avoid multivariate multicollinearity and overfitting in the variable selection procedure (36).

It also requires attention that exacerbation of heart failure is an important driver of deterioration in DCM patients. NYHA class and NT-proBNP on admission had previously been confirmed to be associated with an increased risk of major adverse cardiovascular events (MACE) in DCM patients (37, 38). They are widely used by several models of heart failure to stratify risk and predict prognosis. For this reason, NT-proBNP and NYHA class need to be considered, as we did in our study. In our nomogram model, NYHA class and NT-proBNP were independent predictors of unplanned ICU admissions.

In our study, we found that the level of neutrophils was high in DCM inpatients who were unplanned for ICU admission. Previous studies have shown that inflammation plays a very important role in the development of DCM (39), and neutrophils as an index of inflammation was closely associated with the severity of heart failure in DCM patients. Evidence of inflammatory infiltration has also been found in myocardial biopsy samples from DCM patients (40). Moreover, neutrophils activation may accelerate disease progression in DCM by promoting fibrosis in the myocardium (41). In this study, we found that elevated neutrophil count was an important marker of deterioration and unplanned ICU admission in DCM inpatients. Similarly, risk stratification in NEWS was improved by including the neutrophil count measured at hospital admission, and these improvements were replicated across several different studies (42). Redfern et al. recommended routinely collected blood tests combined with vital signs to assess unplanned ICU admissions (43). Therefore, we included neutrophils in the prediction model we constructed, which was a feature of our model.

The GWTG-HF risk score is a risk assessment tool commonly used to predict in-hospital mortality in patients with heart failure (44). In our patients, the GWTG-HF risk score is also useful as a tool for estimating unplanned ICU admissions in DCM patients (OR: 1.04; 95% CI: 1.02–1.06; *P *< 0.001). We compared the diagnostic efficacy of our nomogram and GWTG-HF risk score using ROC curve analysis. Ultimately, our model performs much better (Figure 8). Hence, it is reasonable to believe that our model has the potential to be a useful tool for evaluating unplanned ICU admissions for DCM inpatients.

Our study also has some limitations. First, this is a single-center and retrospective study, and some patients with incomplete data were also excluded, leading to selective bias. Therefore, multicenter and prospective studies are still necessary to improve the accuracy and applicability of the model. Second, we lacked data needed to externally validate our risk prediction model, so external validation of other clinical research centers is still needed to verify the predictive effect of our nomogram. Third, the population of our study was restricted to DCM inpatients, which also limited its application. A risk assessment study of unplanned ICU admissions for outpatients with DCM will be our next task. In addition, the course of DCM is a dynamic evolutionary process, and a single cross-sectional analysis cannot comprehensively assess the prognosis of DCM, which requires close observation and long-term follow-up, as well as timely adjustment of the treatment plan.

## Conclusion

We developed and validated a new nomogram to predict the risk for unplanned ICU admission in DCM patients, based on six easily accessible independent risk variables. The nomogram may assist physicians in identifying individuals at high unplanned ICU admission risk for DCM inpatients.

## Data Availability

The raw data supporting the conclusions of this article will be made available by the authors, without undue reservation.
